# (3a*S*,7a*S*)-5-[(*S*)-3,3,3-Trifluoro-2-meth­oxy-2-phenyl­propano­yl]-2,3,4,5,6,7-hexa­hydro-1*H*-pyrrolo[3,4-*c*]pyridin-3(2*H*)-one monohydrate

**DOI:** 10.1107/S1600536809053331

**Published:** 2009-12-16

**Authors:** Huichun Zhu, Michael B. Plewe, Arnold L. Rheingold, Curtis Moore, Alex Yanovsky

**Affiliations:** a Pfizer Global Research and Development, La Jolla Labs, 10770 Science Center Drive, San Diego, CA 92121, USA; b Department of Chemistry and Biochemistry, University of California, San Diego, 9500 Gilman Drive, La Jolla, CA 92093, USA

## Abstract

*rac*-Benzyl 3-oxohexa­hydro-1*H*-pyrrolo[3,4-*c*]pyridine-5(6*H*)-carboxyl­ate was separated by chiral chromatography, and one of the enanti­omers ([α]^22^_D_ = +10°) was hydrogenated in the presence of Pd/C in methanol, producing octa­hydro-3*H*-pyrrolo[3,4-*c*]pyridin-3-one. The latter was reacted with (2*R*)-3,3,3-trifluoro-2-meth­oxy-2-phenyl­propanoyl chloride [(*R*)-(−)-Mosher acid chloride], giving rise to the title compound, C_17_H_19_F_3_N_2_O_3_·H_2_O. The present structure established the absolute configuration of the pyrrolopiperidine fragment based on the known configuration of the (*R*)-Mosher acid chloride. The piperidine ring has a somewhat distorted chair conformation and is *cis*-fused with the five-membered envelope-shaped ring; the plane of the exocyclic amide bond is approximately orthogonal to the plane of the phenyl ring, making a dihedral angle of 82.31 (3)°. The water mol­ecule acts as an acceptor to the proton of the amino group in an N—H⋯O inter­action, and as a double proton donor in O—H⋯O hydrogen bonds, generating infinite bands along the *a* axis.

## Related literature

For the structures of compounds with a similar bicyclic fragment, see: Kim *et al.* (2007 ▶); Arnott *et al.* (2006 ▶); Altomare *et al.* (1995 ▶). For the general synthesis method, see: von Dob­eneck & Hansen (1972 ▶).
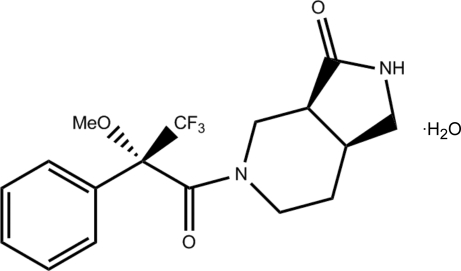


## Experimental

### 

#### Crystal data

C_17_H_19_F_3_N_2_O_3_·H_2_O
*M* *_r_* = 374.36
Orthorhombic, 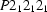

*a* = 8.4870 (4) Å
*b* = 13.7152 (7) Å
*c* = 14.9113 (7) Å
*V* = 1735.69 (15) Å^3^
*Z* = 4
Mo *K*α radiation
μ = 0.12 mm^−1^
*T* = 150 K
0.36 × 0.25 × 0.13 mm

#### Data collection

Bruker APEXII CCD diffractometer
Absorption correction: multi-scan (*SADABS*; Bruker, 2001 ▶) *T*_min_ = 0.957, *T*_max_ = 0.984
23008 measured reflections
4020 independent reflections
3738 reflections with *I* > 2σ(*I*)
*R*_int_ = 0.034

#### Refinement

*R*[*F* ^2^ > 2σ(*F* ^2^)] = 0.030
*wR*(*F* ^2^) = 0.070
*S* = 1.02
4020 reflections
248 parameters
H atoms treated by a mixture of independent and constrained refinement
Δρ_max_ = 0.22 e Å^−3^
Δρ_min_ = −0.19 e Å^−3^
Absolute structure: Flack (1983 ▶), 1537 Friedel pairs
Flack parameter: 0.0 (5)

### 

Data collection: *APEX2* (Bruker, 2007 ▶); cell refinement: *SAINT* (Bruker, 2007 ▶); data reduction: *SAINT*; program(s) used to solve structure: *SHELXS97* (Sheldrick, 2008 ▶); program(s) used to refine structure: *SHELXL97* (Sheldrick, 2008 ▶); molecular graphics: *SHELXTL* (Sheldrick, 2008 ▶); software used to prepare material for publication: *SHELXTL*.

## Supplementary Material

Crystal structure: contains datablocks global, I. DOI: 10.1107/S1600536809053331/kp2243sup1.cif

Structure factors: contains datablocks I. DOI: 10.1107/S1600536809053331/kp2243Isup2.hkl

Additional supplementary materials:  crystallographic information; 3D view; checkCIF report

## Figures and Tables

**Table 1:** Hydrogen-bond geometry (Å, °)

*D*—H⋯*A*	*D*—H	H⋯*A*	*D*⋯*A*	*D*—H⋯*A*
O1*W*—H1*O*⋯O1	0.88 (2)	1.96 (3)	2.8164 (16)	165 (2)
N1—H1*N*⋯O1*W*^i^	0.933 (19)	2.037 (19)	2.8687 (17)	147.7 (16)
O1*W*—H2*O*⋯O2^i^	0.88 (2)	2.06 (2)	2.9111 (15)	162.8 (19)

Symmetry code: (i) 
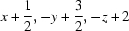
.

**Fig. 1.**
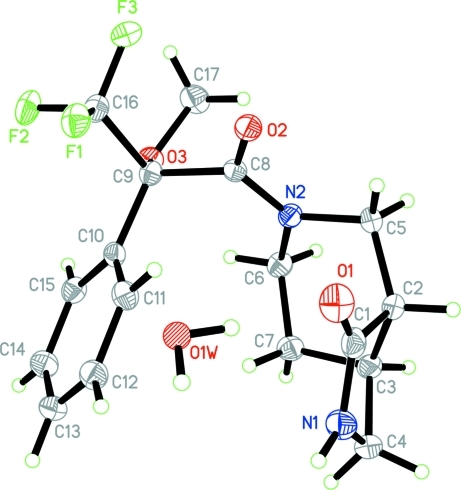
Asymmetric unit of the title compound showing 50% probability displacement ellipsoids and atom numbering scheme. H atoms are drawn as circles of arbitrary small radius.

**Fig. 2.**
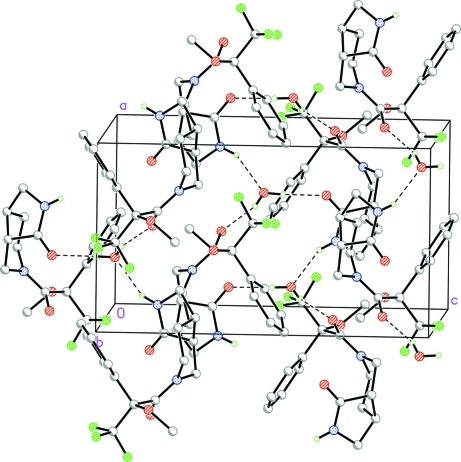
Packing diagram of the title compound viewed down the b axis; hydrogen bonds are shown as dashed lines. H-atoms which do not participate in the hydrogen bonds are omitted.

## supplementary crystallographic information

### Comment

Catalytic hydrogenation of 1*H*-pyrrolo[3,4-*c*]pyridin-3(2*H*)-one (von Dobeneck & Hansen, 1972) with platinum gave *rac*-(3aR*,7aR*)-octahydro-3*H*-pyrrolo[3,4-*c*]-pyridin-3-one which was converted to *rac*-benzyl(3aR*,7aR*)-3-oxooctahydro-5*H*-pyrrolo[3,4-*c*]pyridine-5-carboxylate, and separated into enantiomers by supercritical fluid chiral chromatography on Chiralpak column with 50% methanol-modified CO_2_ as the eluent. The late eluting enantiomer ([α]^22^_D_ = 10°) was hydrogenated in the presence of Pd/C in methanol producing octahydro-3*H*-pyrrolo[3,4-*c*]pyridin-3-one. The latter was reacted with (2*R*)-3,3,3-trifluoro-2-methoxy-2-phenylpropanoyl chloride [(*R*)-(-)-Mosher acid chloride] giving rise to the title compound. The present structure (Fig. 1) established the absolute configuration of the pyrrolopiperidine fragment based on the known configuration at the C9 atom coming from the (*R*)-(-)-Mosher acid chloride (due to a change of the substituent priority of the product, the *S*-configuration is assigned).

The piperidine ring N2/C5/C2/C3/C7/C6 has a somewhat distorted chair conformation with torsion angles of alternating signs and absolute values in the range from 40.5 (2)° to 63.0 (1)°. The *cis*-fused 5-membered ring C1/C2/C3/C4/N1 adopts an envelope shape with the C3 atom displaced by 0.540 (2) Å from the C1/N1/C4/C2 plane which holds to within 0.01 Å. The overall conformation of pyrrolopiperidine fragment is similar to that observed in some of the previously studied compounds with analogous groups (Kim *et al.*, 2007), however the conformation of the rings, especially of the piperidine part, in other related molecules (Arnott *et al.*, 2006; Altomare *et al.*, 1995) is varying broadly depending on the nature of the substituents in the cycle. The amide bond plane (passing through C5/C6/C8/N2/O2/C9 atoms and planar within 0.03 Å) is approximately orthogonal to the plane of the phenyl ring, corresponding dihedral angle being 82.31 (3)°. Water molecule acts as  an acceptor of the HN1 group and as a double acceptor in O-H···O interactions generating hydrogen bond network (Table 1, Fig. 2).

### Experimental

*rac*-(3aR*,7aR*)-Octahydro-3*H*-pyrrolo[3,4-*c*]-pyridin-3-one hydrochloride. A 1.0 *L* Parr shaker bottle was charged with 1,2-dihydropyrrolo[3,4-*c*]pyridin-3-one (68.0 g, 507.5 mmol), acetic acid (400 ml) and PtO_2_ (6.8 g, 10% dry) catalyst (von Dobeneck & Hansen, 1972). A hydrogenation was performed 4 h at 40 psi. The reaction mixture was filtered through celite, and the solvent removed *in vacuo* to furnish an oil. HCl in 1,4-dioxane (4.0 *M*, 126.9 ml) was introduced into the flask at 0°C through an addition funnel. The solvent was then evaporated to dryness, ether (1 *L*) was added, and the mixture was stirred and filtered to yield 65.1 g (73%) of pale yellow solid. ^1^H NMR (300 MHz, DMSO-d_6_) δ 1.50 (m, 1H), 1.82 (m, 1H). 2.45–2.55 (3*H*), 2.64 (m, 1H), 2.82 (m, 2H), 3.06 (m, 2H), 7.88 (s, 1H), 8.33 (brs, 1H), 9.42 (brs, 1H). LC—MS m/*z*: 141.2 (*M*+H)^+^.

*rac*-Benzyl (3aR*,7aR*)-3-oxooctahydro-5*H*-pyrrolo[3,4-*c*]pyridine-5- carboxylate. To *rac*-(3aR*, 7aR*)-octahydro-3*H*-pyrrolo[3,4-*c*]-pyridin-3-one hydrochloride (45.0 g, 0.25 mol) in THF (300 ml) was added K_2_CO_3_ (95.4 g, 0.69 mol), benzyl chloroformate (47.8 g, 0.28 mol), and water (300 ml). The resulting mixture was stirred for 18 h at room temperature. The organic layer was separated, and the aqueous layer was extracted with ethyl acetate (3 *x* 300 ml). The combined organic extracts were dried (Na_2_SO_4_) and concentrated. The residue was recrystallized from heptane to give 68.6 g (99%) white crystals. LC—MS (APCI) m/*z*: 275.1 (*M*+H)+. ^1^H NMR (300 MHz, MeOD) δ 1.37 - 1.56 (m, 1H), 1.75 - 1.93 (m, 1H), 2.45 - 2.73 (m, 2H), 2.86 - 3.08 (m, 2H), 3.24 (d, J = 4.90 Hz, 1H), 3.48 (dd, J = 9.98, 6.22 Hz, 1H), 3.76 - 3.95 (m, 1H), 4.17 - 4.44 (m, 1H), 4.99 - 5.21 (m, 2H), 7.15 - 7.51 (m, 5H). LC—MS (APCI) m/*z*: 275.1 (*M*+H)^+^. The racemate (26 g) was separated into enantiomers using supercritical fluid chromatography on a Chiralpak(*R*) AS—H, 250 *x* 21 mm, 5µ column at 308 K at 100 bar isobaric pressure using 50% methanol-modified CO_2_ and a flow rate of 50 ml/min.

Benzyl (3aR,7aR)-3-oxooctahydro-5*H*-pyrrolo[3,4-*c*]pyridine-5-carboxylate: 12.1 g (46%), *R*_f_ = 0.87, ee: 100%, LC–MS (APCI) *m*/*z*: 275.3 (*M*+H)^+^. [α]^22^_D_ = -13.5° (C=0.006 g/ml, MeOH).

Benzyl (3aS,7aS)-3-oxooctahydro-5*H*-pyrrolo[3,4-*c*]pyridine-5-carboxylate: 12.1 g (46%), *R*_f_ = 1.88, ee: 100%. LC—MS (APCI) m/*z*: 275.3. [α]^22^_D_ = +10.1° (c 0.007 g/ml, MeOH).

(3aS,7aS)-Octahydro-3*H*-pyrrolo[3,4-*c*]pyridin-3-one. To a solution of benzyl (3aR,7aR)-3-oxooctahydro-5*H*-pyrrolo[3,4-*c*]pyridine-5-carboxylate (500 mg, 1.82 mmol) in MeOH (5 ml) was added Pd/C (25 mg, 10 wt. % on activated carbon). The mixture was stirred for 18 h at room-temperature with a hydrogen balloon. The Pd/C was filtered off, and the filtrate was concentrated. Yield: 240 mg (95%). [α]^22^_D_ = -25.9° (c 0.004 g/ml, MeOH).

(3aS,7aS)-5-((*S*)-3,3,3-trifluoro-2-methoxy-2-phenylpropanoyl)hexahydro-1*H*- pyrrolo[3,4-*c*]pyridin-3(2*H*)-one. To a solution of (3aS,7aS)-octahydro-3*H*-pyrrolo[3,4-*c*]pyridin-3-one (180 mg, 1.28 mmol) in DMF (2 ml) was added DIEA (498 mg, 1.28 mmol). The mixture was stirred at 0°C for 5 min. (2*R*)-3,3,3-trifluoro-2-methoxy-2- phenylpropanoyl chloride (Mosher acid chloride, 324 mg, 1.28 mmol) was added at 273 K. The reaction mixture was slowly warmed up to room-temperature and stirred for 10 min at room temperature. The product was purified by flash chromatography (ISCO, 10% MeOH in DCM, UV collect all) to afford 347 mg (76%) of white solid. ^1^H NMR (300 MHz, MeOD) δ 0.18 - 0.37 (m, 1H), 1.09 - 1.23 (m, 1H), 2.47 (dq, J = 11.56, 5.94 Hz, 1H), 2.55 - 2.72 (m, 2H), 2.85 - 3.12 (m, 2H), 3.37 - 3.54 (m, 1H), 3.59 - 3.79 (m, 3H), 3.84 - 3.99 (m, 1H), 4.94 - 5.06 (m, 1H), 7.38 - 7.44 (m, 2H), 7.44 - 7.57 (m, 3H). LC—MS (APCI) m/*z*: 357.2 (*M*+H)^+^. [α]^22^_D_ = -57.9° (c 0.007 g/ml, MeOH). Crystals of the subject compound were grown by heating the sample in a 353 K sand bath for one hour and then allowing it to cool and sit at room temperature for several days.

### Refinement

All H atoms, bonded to C, were placed geometrically (C—H 0.95, 0.98, 0.99 and 1.00 Å for aromatic, methyl, methylene and methine groups respectively) and included in the refinement in riding motion approximation with *U*_iso_(H) set to 1.2*U*_eq_(C) [1.5*U*_eq_(C) for methyl H atoms]. The H atoms at the N and O atoms were located in the difference map and refined isotropically [N—H 0.93 (2) Å; both O—H 0.88 (2) Å]. Even though there are no atoms with *Z*>Si, taking into account the chirality of the molecule, we chose not to merge the Friedel pairs. The Flack parameter, as one would expect, is inconclusive, and the absolute configuration was assigned on the basis of the known chirality of the starting material [(*R*)-(-)-Mosher acid chloride].

### Crystal data

**Table:**

C_17_H_19_F_3_N_2_O_3_·H_2_O	*F*(000) = 784
*M**_r_* = 374.36	*D*_x_ = 1.433 Mg m^−^^3^
Orthorhombic, *P*2_1_2_1_2_1_	Mo *K*α radiation, λ = 0.71073 Å
Hall symbol: P 2ac 2ab	Cell parameters from 7669 reflections
*a* = 8.4870 (4) Å	θ = 2.7–27.4°
*b* = 13.7152 (7) Å	µ = 0.12 mm^−^^1^
*c* = 14.9113 (7) Å	*T* = 150 K
*V* = 1735.69 (15) Å^3^	Block, colourless
*Z* = 4	0.36 × 0.25 × 0.13 mm

### Data collection

**Table:**

Bruker APEX CCD diffractometer	4020 independent reflections
Radiation source: fine-focus sealed tube	3738 reflections with *I* > 2σ(*I*)
graphite	*R*_int_ = 0.034
φ and ω scans	θ_max_ = 28.4°, θ_min_ = 2.0°
Absorption correction: multi-scan (*SADABS*; Bruker, 2001)	*h* = −11→10
*T*_min_ = 0.957, *T*_max_ = 0.984	*k* = −17→18
23008 measured reflections	*l* = −19→18

### Refinement

**Table:**

Refinement on *F*^2^	Secondary atom site location: difference Fourier map
Least-squares matrix: full	Hydrogen site location: inferred from neighbouring sites
*R*[*F*^2^ > 2σ(*F*^2^)] = 0.030	H atoms treated by a mixture of independent and constrained refinement
*wR*(*F*^2^) = 0.070	*w* = 1/[σ^2^(*F*_o_^2^) + (0.0343*P*)^2^ + 0.299*P*] where *P* = (*F*_o_^2^ + 2*F*_c_^2^)/3
*S* = 1.02	(Δ/σ)_max_ = 0.001
4020 reflections	Δρ_max_ = 0.22 e Å^−^^3^
248 parameters	Δρ_min_ = −0.19 e Å^−^^3^
0 restraints	Absolute structure: Flack (1983), 1537 Friedel pairs
Primary atom site location: structure-invariant direct methods	Flack parameter: 0.0 (5)

### Special details

**Table:**

Geometry. All e.s.d.'s (except the e.s.d. in the dihedral angle between two l.s. planes) are estimated using the full covariance matrix. The cell e.s.d.'s are taken into account individually in the estimation of e.s.d.'s in distances, angles and torsion angles; correlations between e.s.d.'s in cell parameters are only used when they are defined by crystal symmetry. An approximate (isotropic) treatment of cell e.s.d.'s is used for estimating e.s.d.'s involving l.s. planes.
Refinement. Refinement of *F*^2^ against ALL reflections. The weighted *R*-factor *wR* and goodness of fit *S* are based on *F*^2^, conventional *R*-factors *R* are based on *F*, with *F* set to zero for negative *F*^2^. The threshold expression of *F*^2^ > σ(*F*^2^) is used only for calculating *R*-factors(gt) *etc*. and is not relevant to the choice of reflections for refinement. *R*-factors based on *F*^2^ are statistically about twice as large as those based on *F*, and *R*- factors based on ALL data will be even larger.

### Fractional atomic coordinates and isotropic or equivalent isotropic displacement parameters (Å^2^)

**Table:**

	*x*	*y*	*z*	*U*_iso_*/*U*_eq_	
C1	0.46490 (18)	0.72722 (9)	0.80771 (10)	0.0226 (3)	
C2	0.43300 (15)	0.68038 (10)	0.71644 (9)	0.0201 (3)	
H2	0.4493	0.7315	0.6695	0.024*	
C3	0.56770 (15)	0.60584 (10)	0.70764 (10)	0.0216 (3)	
H3	0.5959	0.5953	0.6433	0.026*	
C4	0.70183 (16)	0.65726 (11)	0.75757 (10)	0.0255 (3)	
H4A	0.7755	0.6096	0.7846	0.031*	
H4B	0.7612	0.7012	0.7172	0.031*	
C5	0.26691 (16)	0.64039 (10)	0.70513 (9)	0.0201 (3)	
H5A	0.1896	0.6895	0.7259	0.024*	
H5B	0.2466	0.6274	0.6409	0.024*	
C6	0.36089 (16)	0.47410 (9)	0.73187 (9)	0.0202 (3)	
H6A	0.3522	0.4596	0.6670	0.024*	
H6B	0.3380	0.4136	0.7656	0.024*	
C7	0.52719 (16)	0.50895 (9)	0.75338 (10)	0.0217 (3)	
H7A	0.6036	0.4587	0.7339	0.026*	
H7B	0.5378	0.5167	0.8191	0.026*	
C8	0.13686 (15)	0.54710 (9)	0.82186 (9)	0.0164 (2)	
C9	0.12702 (15)	0.45319 (9)	0.87997 (9)	0.0170 (3)	
C10	0.27529 (15)	0.44736 (9)	0.93844 (9)	0.0180 (3)	
C11	0.33668 (16)	0.53289 (10)	0.97544 (9)	0.0210 (3)	
H11	0.2841	0.5932	0.9660	0.025*	
C12	0.47440 (17)	0.53028 (10)	1.02599 (9)	0.0236 (3)	
H12	0.5161	0.5887	1.0507	0.028*	
C13	0.55050 (16)	0.44225 (10)	1.04025 (10)	0.0245 (3)	
H13	0.6450	0.4404	1.0744	0.029*	
C14	0.48909 (16)	0.35704 (10)	1.00488 (10)	0.0245 (3)	
H14	0.5409	0.2967	1.0155	0.029*	
C15	0.35139 (16)	0.35918 (9)	0.95366 (9)	0.0204 (3)	
H15	0.3099	0.3006	0.9293	0.025*	
C16	−0.01874 (16)	0.45986 (9)	0.94228 (9)	0.0211 (3)	
C17	0.00234 (19)	0.36490 (10)	0.75741 (10)	0.0276 (3)	
H17A	−0.1051	0.3590	0.7809	0.041*	
H17B	0.0251	0.3095	0.7180	0.041*	
H17C	0.0119	0.4258	0.7234	0.041*	
N1	0.61752 (15)	0.71226 (9)	0.82667 (9)	0.0264 (3)	
N2	0.24673 (13)	0.55010 (8)	0.75654 (7)	0.0180 (2)	
O1	0.36798 (13)	0.77208 (7)	0.85314 (7)	0.0301 (2)	
O2	0.04938 (11)	0.61587 (6)	0.83902 (6)	0.0208 (2)	
O3	0.11272 (11)	0.36562 (6)	0.83085 (6)	0.0201 (2)	
O1W	0.36093 (13)	0.76305 (8)	1.04178 (8)	0.0273 (2)	
F1	−0.00782 (10)	0.52989 (5)	1.00457 (5)	0.02504 (18)	
F2	−0.03398 (10)	0.37511 (6)	0.98714 (6)	0.0283 (2)	
F3	−0.15420 (9)	0.47343 (6)	0.89841 (6)	0.02748 (19)	
H1O	0.359 (3)	0.7767 (16)	0.9843 (17)	0.059 (7)*	
H2O	0.424 (3)	0.8062 (16)	1.0675 (14)	0.052 (6)*	
H1N	0.665 (2)	0.7341 (13)	0.8794 (13)	0.037 (5)*	

### Atomic displacement parameters (Å^2^)

**Table:**

	*U*^11^	*U*^22^	*U*^33^	*U*^12^	*U*^13^	*U*^23^
C1	0.0274 (7)	0.0175 (6)	0.0230 (7)	−0.0043 (6)	−0.0012 (6)	0.0047 (5)
C2	0.0194 (6)	0.0223 (6)	0.0188 (7)	−0.0023 (5)	0.0006 (5)	0.0046 (5)
C3	0.0174 (6)	0.0292 (7)	0.0181 (7)	−0.0013 (6)	0.0009 (5)	0.0011 (5)
C4	0.0188 (6)	0.0302 (7)	0.0276 (8)	−0.0047 (6)	−0.0033 (5)	0.0056 (6)
C5	0.0195 (6)	0.0225 (6)	0.0183 (7)	−0.0003 (5)	−0.0010 (5)	0.0053 (5)
C6	0.0203 (6)	0.0201 (6)	0.0202 (7)	0.0014 (6)	0.0035 (5)	−0.0028 (5)
C7	0.0194 (7)	0.0225 (6)	0.0231 (7)	0.0030 (5)	0.0017 (5)	−0.0009 (5)
C8	0.0156 (6)	0.0182 (6)	0.0155 (6)	−0.0018 (5)	−0.0023 (5)	0.0001 (5)
C9	0.0185 (6)	0.0159 (5)	0.0166 (7)	−0.0001 (5)	0.0017 (5)	−0.0004 (5)
C10	0.0184 (6)	0.0206 (6)	0.0150 (7)	0.0014 (5)	0.0016 (5)	0.0023 (5)
C11	0.0246 (7)	0.0191 (6)	0.0194 (7)	0.0024 (6)	−0.0010 (5)	0.0001 (5)
C12	0.0256 (7)	0.0252 (6)	0.0199 (7)	−0.0031 (6)	−0.0012 (5)	−0.0013 (5)
C13	0.0181 (6)	0.0326 (7)	0.0229 (8)	0.0013 (6)	−0.0019 (6)	0.0034 (6)
C14	0.0221 (7)	0.0239 (6)	0.0275 (8)	0.0058 (6)	0.0004 (6)	0.0031 (5)
C15	0.0215 (6)	0.0177 (6)	0.0221 (7)	0.0004 (5)	0.0027 (6)	0.0008 (5)
C16	0.0220 (6)	0.0204 (6)	0.0210 (7)	0.0006 (6)	0.0035 (5)	0.0023 (5)
C17	0.0303 (7)	0.0235 (6)	0.0290 (8)	−0.0063 (6)	−0.0092 (6)	−0.0017 (6)
N1	0.0286 (6)	0.0264 (6)	0.0241 (7)	−0.0067 (5)	−0.0068 (5)	−0.0001 (5)
N2	0.0174 (5)	0.0188 (5)	0.0177 (6)	−0.0010 (5)	0.0009 (4)	0.0003 (4)
O1	0.0373 (6)	0.0251 (5)	0.0280 (6)	−0.0004 (5)	0.0005 (5)	−0.0024 (4)
O2	0.0187 (4)	0.0200 (4)	0.0238 (5)	0.0030 (4)	0.0021 (4)	0.0028 (4)
O3	0.0211 (5)	0.0182 (4)	0.0211 (5)	−0.0020 (4)	−0.0017 (4)	−0.0023 (4)
O1W	0.0307 (6)	0.0256 (5)	0.0256 (6)	−0.0068 (5)	0.0051 (5)	−0.0049 (4)
F1	0.0294 (4)	0.0237 (4)	0.0220 (4)	0.0044 (3)	0.0064 (3)	−0.0012 (3)
F2	0.0337 (5)	0.0227 (4)	0.0286 (5)	−0.0006 (4)	0.0105 (4)	0.0069 (3)
F3	0.0172 (4)	0.0350 (5)	0.0302 (5)	−0.0007 (4)	0.0029 (3)	0.0035 (4)

### Geometric parameters (Å, °)

**Table:**

C1—O1	1.2305 (18)	C9—O3	1.4120 (15)
C1—N1	1.3416 (19)	C9—C10	1.5330 (18)
C1—C2	1.529 (2)	C9—C16	1.5498 (18)
C2—C5	1.5218 (18)	C10—C15	1.3898 (18)
C2—C3	1.5392 (18)	C10—C11	1.3971 (19)
C2—H2	1.0000	C11—C12	1.3913 (19)
C3—C4	1.5321 (19)	C11—H11	0.9500
C3—C7	1.5328 (19)	C12—C13	1.3857 (19)
C3—H3	1.0000	C12—H12	0.9500
C4—N1	1.464 (2)	C13—C14	1.384 (2)
C4—H4A	0.9900	C13—H13	0.9500
C4—H4B	0.9900	C14—C15	1.3964 (19)
C5—N2	1.4664 (16)	C14—H14	0.9500
C5—H5A	0.9900	C15—H15	0.9500
C5—H5B	0.9900	C16—F3	1.3357 (16)
C6—N2	1.4699 (17)	C16—F1	1.3393 (15)
C6—C7	1.5242 (19)	C16—F2	1.3473 (14)
C6—H6A	0.9900	C17—O3	1.4412 (16)
C6—H6B	0.9900	C17—H17A	0.9800
C7—H7A	0.9900	C17—H17B	0.9800
C7—H7B	0.9900	C17—H17C	0.9800
C8—O2	1.2273 (15)	N1—H1N	0.933 (19)
C8—N2	1.3490 (17)	O1W—H1O	0.88 (2)
C8—C9	1.5546 (17)	O1W—H2O	0.88 (2)
			
O1—C1—N1	127.29 (14)	O3—C9—C16	107.02 (10)
O1—C1—C2	125.57 (13)	C10—C9—C16	108.50 (10)
N1—C1—C2	107.12 (13)	O3—C9—C8	114.85 (10)
C5—C2—C1	114.46 (12)	C10—C9—C8	108.42 (10)
C5—C2—C3	116.05 (11)	C16—C9—C8	109.16 (10)
C1—C2—C3	102.91 (11)	C15—C10—C11	119.53 (12)
C5—C2—H2	107.7	C15—C10—C9	121.31 (12)
C1—C2—H2	107.7	C11—C10—C9	119.14 (11)
C3—C2—H2	107.7	C12—C11—C10	120.38 (12)
C4—C3—C7	110.46 (12)	C12—C11—H11	119.8
C4—C3—C2	101.82 (11)	C10—C11—H11	119.8
C7—C3—C2	111.79 (11)	C13—C12—C11	119.80 (12)
C4—C3—H3	110.8	C13—C12—H12	120.1
C7—C3—H3	110.8	C11—C12—H12	120.1
C2—C3—H3	110.8	C14—C13—C12	120.12 (13)
N1—C4—C3	102.48 (11)	C14—C13—H13	119.9
N1—C4—H4A	111.3	C12—C13—H13	119.9
C3—C4—H4A	111.3	C13—C14—C15	120.39 (13)
N1—C4—H4B	111.3	C13—C14—H14	119.8
C3—C4—H4B	111.3	C15—C14—H14	119.8
H4A—C4—H4B	109.2	C10—C15—C14	119.77 (12)
N2—C5—C2	110.77 (11)	C10—C15—H15	120.1
N2—C5—H5A	109.5	C14—C15—H15	120.1
C2—C5—H5A	109.5	F3—C16—F1	107.42 (10)
N2—C5—H5B	109.5	F3—C16—F2	106.30 (10)
C2—C5—H5B	109.5	F1—C16—F2	106.33 (10)
H5A—C5—H5B	108.1	F3—C16—C9	113.68 (11)
N2—C6—C7	109.59 (10)	F1—C16—C9	113.76 (11)
N2—C6—H6A	109.8	F2—C16—C9	108.86 (10)
C7—C6—H6A	109.8	O3—C17—H17A	109.5
N2—C6—H6B	109.8	O3—C17—H17B	109.5
C7—C6—H6B	109.8	H17A—C17—H17B	109.5
H6A—C6—H6B	108.2	O3—C17—H17C	109.5
C6—C7—C3	112.70 (11)	H17A—C17—H17C	109.5
C6—C7—H7A	109.1	H17B—C17—H17C	109.5
C3—C7—H7A	109.1	C1—N1—C4	113.73 (12)
C6—C7—H7B	109.1	C1—N1—H1N	123.0 (12)
C3—C7—H7B	109.1	C4—N1—H1N	123.2 (12)
H7A—C7—H7B	107.8	C8—N2—C5	118.94 (11)
O2—C8—N2	123.04 (12)	C8—N2—C6	127.93 (11)
O2—C8—C9	119.21 (11)	C5—N2—C6	113.02 (10)
N2—C8—C9	117.70 (11)	C9—O3—C17	117.12 (10)
O3—C9—C10	108.74 (10)	H1O—O1W—H2O	107.0 (19)
			
O1—C1—C2—C5	−33.31 (19)	C10—C11—C12—C13	−0.4 (2)
N1—C1—C2—C5	148.36 (12)	C11—C12—C13—C14	−0.5 (2)
O1—C1—C2—C3	−160.12 (13)	C12—C13—C14—C15	0.8 (2)
N1—C1—C2—C3	21.55 (13)	C11—C10—C15—C14	−0.67 (19)
C5—C2—C3—C4	−158.45 (12)	C9—C10—C15—C14	177.92 (12)
C1—C2—C3—C4	−32.67 (13)	C13—C14—C15—C10	−0.3 (2)
C5—C2—C3—C7	−40.53 (16)	O3—C9—C16—F3	−68.07 (13)
C1—C2—C3—C7	85.25 (13)	C10—C9—C16—F3	174.76 (11)
C7—C3—C4—N1	−86.77 (13)	C8—C9—C16—F3	56.79 (14)
C2—C3—C4—N1	32.10 (14)	O3—C9—C16—F1	168.56 (10)
C1—C2—C5—N2	−73.86 (14)	C10—C9—C16—F1	51.39 (13)
C3—C2—C5—N2	45.83 (16)	C8—C9—C16—F1	−66.59 (13)
N2—C6—C7—C3	−56.07 (15)	O3—C9—C16—F2	50.20 (13)
C4—C3—C7—C6	157.69 (12)	C10—C9—C16—F2	−66.97 (13)
C2—C3—C7—C6	45.08 (15)	C8—C9—C16—F2	175.06 (10)
O2—C8—C9—O3	129.59 (12)	O1—C1—N1—C4	−178.89 (14)
N2—C8—C9—O3	−52.90 (15)	C2—C1—N1—C4	−0.60 (15)
O2—C8—C9—C10	−108.58 (13)	C3—C4—N1—C1	−20.66 (15)
N2—C8—C9—C10	68.93 (14)	O2—C8—N2—C5	1.99 (19)
O2—C8—C9—C16	9.44 (16)	C9—C8—N2—C5	−175.41 (11)
N2—C8—C9—C16	−173.05 (11)	O2—C8—N2—C6	177.85 (12)
O3—C9—C10—C15	−14.71 (16)	C9—C8—N2—C6	0.44 (19)
C16—C9—C10—C15	101.35 (14)	C2—C5—N2—C8	119.23 (13)
C8—C9—C10—C15	−140.21 (12)	C2—C5—N2—C6	−57.22 (14)
O3—C9—C10—C11	163.89 (11)	C7—C6—N2—C8	−113.09 (14)
C16—C9—C10—C11	−80.05 (14)	C7—C6—N2—C5	62.97 (14)
C8—C9—C10—C11	38.39 (15)	C10—C9—O3—C17	−166.32 (11)
C15—C10—C11—C12	1.0 (2)	C16—C9—O3—C17	76.67 (13)
C9—C10—C11—C12	−177.60 (12)	C8—C9—O3—C17	−44.66 (15)

### Hydrogen-bond geometry (Å, °)

**Table:**

*D*—H···*A*	*D*—H	H···*A*	*D*···*A*	*D*—H···*A*
O1W—H1O···O1	0.88 (2)	1.96 (3)	2.8164 (16)	165 (2)
N1—H1N···O1W^i^	0.933 (19)	2.037 (19)	2.8687 (17)	147.7 (16)
O1W—H2O···O2^i^	0.88 (2)	2.06 (2)	2.9111 (15)	162.8 (19)

Symmetry codes: (i) *x*+1/2, −*y*+3/2, −*z*+2.
